# How the social media impact women’s psychological well-being in the patriarchal structure? The moderating effect of social capital

**DOI:** 10.1186/s12889-024-18013-y

**Published:** 2024-02-23

**Authors:** Liu He, Amira Firdaus, Jiankun Gong, Nasrullah Dharejo, Iffat Ali Aksar

**Affiliations:** 1 Media art research center, Jiangxi Institute of Fashion Technology, Nanchang, China; 2 Department of Media and Communication Studies, University of Malaya, Kuala Lumpur, Malaysia; 3 International college, Krirk University, Bangkok, 10220 Thailand; 4 Media and Communication Sukkur IBA University, Karachi, Pakistan; 5 School of Communication, Xiamen University, Sepang, Malaysia

**Keywords:** Social media use, Women, Psychological well-being, Online social capital, Pakistan

## Abstract

**Background:**

Despite technological, political and economic progress, Pakistan is still a traditionally patriarchal society, and cultural norms curb women’s freedom of socialization, which contributes to poor mental health. The digital technology spaces are rampant with male dominance, and offline cultural behaviours are replicated. Therefore, the current research in Pakistan intends to focus solely on women, their social media uses and the consequent impact on their psychological well-being. Furthermore, the mediation role of social capital is explored, which is linked to women’s socialization. In virtual communication, women can expand their connection or remain limited to known people.

**Methods:**

An online survey collected 240 responses from women social media users. The questionnaire was divided into demographics, social media use patterns like access, online time, frequency of use, social media uses, online social capital and psychological well-being. The obtained responses were statistically analyzed using Smart PLS.

**Results:**

Pakistani women use social media extensively; however, their uses are culturally influenced. The women use social media and socialize online but do not openly disclose their personalities and emotions to extend the connection. They seek information only from acquaintances and do not trust newly developed online contacts. Therefore, the mediation role of bonding social capital is significant, referring to the importance of close ties and trust in psychological well-being. Though virtual spaces provide an opportunity for bridging social capital, women use social media for socialization; however, it doesn’t contribute to women’s psychological well-being.

**Conclusion:**

Despite the higher penetration of digital technologies, cultural power still rules in developing countries like Pakistan. Social media uses are gender- and culturally specific, contributing to psychological well-being and developing social capital. The results from Pakistani society recommend ensuring a secure digital experience for women to get maximum benefits from social media and enhance their psychological well-being.

## Introduction

Regarding access to technology, the digital divide is still a harsh reality of modern life. United Nations and its partner organizations like UNESCO, Organisation for Economic Co-operation and Development, still report a digital divide gap. The gap is mainly wider in traditional societies, where gender disparities still rule and name the gender digital divide. Thus, regional culture, social structure, norms and gender status impact how technologies are adopted and their effects [1–3].

The development and inclusion of technologies in Western societies transformed their social, cultural and economic fields and improved peoples’ well-being. Inspired by the transformational changes in human and social sectors, the scholars suggested the inclusion of technologies for the global south as well to eradicate gender and cultural barriers and improve people’s lives [4].

However, the ground realities of the global south differ from the Global North in terms of political, economic, social and cultural aspects. The democratic political system, digitalization, high economic indexes, policies and Westernization cannot change century-old gender segregation, which is contributing to the gender digital divide [5, 6]. Whether it is offline disparities or online inequalities, women are the sufferer. In the offline environment, discriminatory behaviour and low status negatively influence their well-being, either emotionally or psychologically. Worldwide, psychological disorders, like depression, stress, anxiety and low self-esteem are observed more among women [7]. Similarly, access to technology among women is far less than men, and women are minorities in the online environment. Thus, for women, the offline and online experiences are not different.

However, changing new media landscape worldwide, particularly in developing Asian countries like Pakistan, offer an ideal ground to explore the effects of technologies on well-being and see whether there is any change in offline and online culture. There is a paucity of academic research to examine the psychological impacts of new media from cultural and gender perspectives [8]. Therefore, academic efforts are needed to align gender and culture and see how social media is contributing to psychological well-being among women, who are living in a male-dominated society. The comparative studies between males and females have identified differences in uses among both gender, however, there is a wide gap in understanding how a woman in a patriarchal culture perceive the psychological impact of social media and how social media help women in socialization, which is restricted culturally [9].

Additionally, the studies established the association between social media use and social capital, however, there is a gap in understanding the mediation role of social capital, specifically in developing countries [10]. Furthermore, no study has yet examined the mediation effect of social capital between social media use and psychological well-being among women. Therefore, the current study intends to explore the impact of social media use and women’s psychological well-being through social capital.

## Study context

Women in Pakistan suffer from a high rate of mental and psychological illness due to domestic violence, financial dependency, illiteracy, and a patriarchal setup which dominates all fields of life [11].

Likewise, Social media usage is comparable to developed nations, however, the use of social media among women is limited. Pakistani men rule online spaces, creating an unsafe online environment for women and causing online abuse [12]. In Pakistan, there is a wide digital gender gap of 73% males and 27% females. However, scholars advocated the inclusion of technologies to diminish gender disparities and bridge the digital gap [13] and stress providing equal socialization opportunities for better well-being. Specifically, meaningful use of technology is required in developing countries to empower the masses [14]. However, an oppressive society believes in gender inequities and hinders women’s growth. These oppressive forces and gender-discriminatory behaviours reduce women’s liberty offline and online [15], which lowers women’s psychological well-being [16]. (So, resultantly women don’t make much progress in their life.

Therefore, there is a dire academic need to collect evidence and present the actual impacts of technologies on social structure and consequent well-being. Thus, the study serves two purposes. One is to reveal the use of social media among women, who are always described as the vulnerable victim of cultural powers, and the second is to examine the impacts of social media on their psychological well-being. The patriarchal culture mainly designates women’s low status and restricts their socialization. Therefore, by adopting social capital and psychological well-being theoretical approaches, the study stated two research questions to answer (a) whether women are free from the patriarchal cage to extend their socialization due to social media? (b) To what extent the freedom of online social capital contributes to their psychological well-being?

## Theoretical framework

The current research is grounded in three theoretical assumptions: Uses and Gratification theory (U&GT) [17], social capital [18] and psychological well-being [19]. Since the study is about new media, commonly known as social media, the U&GT approach is adopted to understand the uses of social media among the sample (women) in Pakistan. The second concept, social capital, is employed as Putnam [18] described it as the “strength of ties” and categorized it as “bonding” and “bridging.” The bonding social capital indicates a “closed structure of close knitted-community, beneficial for close relations, and provides social and emotional support.” The “bridging social capital” is an “open structure where people interact beyond their network, gain and exchange information and make outside ties.” Though the conceptual and theoretical ambiguities related to the term “social capital” still exist [20]. However, the conceptualization of social capital in bonding and bridging relates to current research. The patriarchal culture keeps little room for women to go beyond their close relationships and extend their network. Therefore, the study assumes that social media is not setting boundaries for women and helping them to expand their socialization and make new connections. Since the offline culture believes in “*Chadar and Chaar Dewari*” (veil and four walls), social media allow them to go beyond culturally constructed four walls. Conversely, the study also intends to verify whether women use digital media technologies to bridge social capital or are still limited to their close ties and strengthen their bonding social capital. Studies have revealed the positive contribution of social media in building social capital [21], integrating communities and people [22], and strengthening networks and socialization [23]. Furthermore, recent scholars have advocated the role of social media in developing social capital among women for sharing knowledge and empowerment [24]. However, these evidences are from developed Western countries, however, can we generalize these findings to culturally conservative societies? Specifically relating to women, can we assume that social media offer equal opportunities to women in developing social capital and extending their socialization? Therefore, the current is carried out to get women’s insight from patriarchal culture. For example, does social media use helping women in Pakistan to develop social capital? Do women take advantage of social media to extend their connections beyond their known circle? Do women have more trust in online contacts or the offline cultural experience, keep them in a limited known circle of communication where they have a trust? Hence, the study categorizes social capital as bonding and bridging social capital and adopts the well-validated ISCS by Willaims [25] to explore the relationship between social media use and social capital among women in a patriarchal cultural context.

Lastly, the concept of psychological well-being, as defined by Ryff [19] is adopted for the current study. It defined well-being, as, “*not only the absence of illness but the presence of something positive.*” Different humanitarian and social welfare organizations also emphasized well-being and suggested human well-being indices [26], such as “Better Life Index by the Organization for Economic Co-operation and Development (OECD), the Global Well-being Index (GWI), the Happy Planet Index (HPI), the Human Development Index (HDI), the Sustainable Society Index (SSI).” These indices look beyond the GDP measurement, emphasize social and human issues, and suggest further research on well-being [27]. Previously, “happiness and life satisfaction” were interchangeably used for “well-being.” However, according to Ryff [28], “well-being is not limited to happiness and life satisfaction but also highlights “all activities that are necessary for human attitude, behaviour or virtue.” She claimed “happiness” as a short-term “affective well-being” (hedonic approach), but for “sustainable well-being,” the sense of “meaning in life and inner self-realization” was necessary. The “well-being” concept focuses on the “individual’s cognitive representation and good life experience”. The “psychological well-being” model by Ryff emerged and explained all aspects of “individuals’ lifelike development and self-realization” [29]. Six dimensions of psychological well-being:

- Self-acceptance: Acknowledging oneself with potential and limitations.
- Positive relations: The level of significant relations and connection.
- Personal growth: The continued growth and development as a person.
- Purpose in life: Having a purpose in life and understanding meaningful goals.
- Autonomy: The sense of independence and confidence in thought and action.
- Environmental Mastery: The ability to manage complex environments to suit personal needs and values.

Studies in the developed part of the world observed the positive impacts of new media technologies on psychological well-being [30], such as self-acceptance [31]. However, in developing countries, the concept of psychological well-being is at an infant stage [32].

## Literature review

### Social media, online social capital and psychological well-being

The current era of a saturated online environment has resulted in diverse impacts of new media technologies on human behaviour, interaction and socialization [33]. Likewise, academically new media research has evolved and impacts are categorized specifically, such as behavioural, social, emotional, cognitive and psychological. In particular, the psychological impacts are categorised as either positive or negative. The negative impacts are discussed as depression, anxiety, stress, and life dissatisfaction, whereas positive impacts include life satisfaction, happiness and pleasure [34].

The exploration of negative consequences began after a well-cited and pioneer study by Kraut et al. [35], which concluded with the negative effects of the internet. Kraut and his team described that the internet is reducing social interaction and causing harmful effects on psychological well-being. Furthermore, the longitudinal study revealed higher loneliness, stress, and depression among internet users [35]. Resultantly, these findings raised concerns among scholars; a great deal of research was conducted and examined psychological outcomes of new media use, such as “internet addiction, social isolation, cyberbullying, cyber racism, low self-esteem” [36]. Furthermore, spending excessive hours online is “misusing " the internet, and avoiding personal relationships leads to anxiety and depression [37]. Excessive online time decreased face-to-face contact, increased social isolation, sleep loss, stress, emotional animosity, and rude and harmful behaviour, negatively affecting mental health and psychological well-being [38]. Contrary to these findings, many studies showed that social media improves psychological well-being, social support, social cohesion, and quality of life [39, 40]. These studies found that social media engages people and provides chances to improve social lives [41]. Along with the direct impacts of social media on psychological well-being, the moderating and mediating factors are further discussed by scholars. For example, one study indicated moderating effect of gender between social media and psychological well-being [1, 42]. Furthermore, culture moderates the relationship between social media and psychological well-being [43]. Social capital mediates the positive association of social media and subjective well-being [44]. Previously, mediating effects of Social capital was discuused between social media and psychological well-being [45–47]. Recently, the effects of bridging and bonding social capital are explored on the association between social media motives and psychological well-being). With time, several social networking sites have been introduced and gradually online time has increased, therefore, studies are needed to see how social media increases or reduces social capital.

Additionally, the studies are mainly focused on males and females, from developed countries, and there is a lack of empirical research focused on women and culture. Due to the lack of cross-cultural studies, experts suggested interdisciplinary studies on how social media affects social ties and quality of life. The studies concluded that inconsistency of results is not just due to timeframe, sample, age, and socio-economic status, but most importantly due to culture, region, Internet penetration and accessibility, and the availability of devices used to access the Internet [48]. For example, minorities and marginalized groups have new opportunities for networking, idea sharing, and online learning due to the pervasive use of the Internet and social media in emerging nations, which has the potential to increase psychological well-being [49]. Considering the conflicting results and the scarcity of social media studies in developing countries, especially Pakistan, examining social media’s impacts on social capital and women’s psychological well-being is a significant academic addition.

### Conceptual framework and hypotheses development

The literature review identified inconsistent results and the need to expand research to explore the relationships among social media, social capital, and psychological well-being; however, considerable gaps exist in the body of knowledge pertaining to: (a) the specific social media use of women; (b) social media research in developing countries; (c) the real-life digital experiences of women in patriarchal forces; and (d) the mediating role of social capital between social media needs and psychological well-being. In addition, studies have rarely focused on psychological well-being (eudaimonic effects) as an outcome, as most studies measure subjective well-being and life satisfaction. Above all, no study has been conducted to explore the effects of social media use on women’s social capital and psychological well-being in Pakistan.

Therefore, the current study attempted to fill these gap by answering scholars’ calls to expand social media research across diverse contexts and overlook and examined the relationships with psychological well-being by building women’s online social capital in the collectivistic and patriarchal Pakistani society [50].

Based on this framework, the following hypotheses were formulated:

H_1_ Social media use positively influences the psychological well-being of women in Pakistan.

H_2_: Social media use positively influences women’s social capital (bonding social capital and bridging social capital) in Pakistan.

H_3_: Online social capital (bonding and bridging social capital) positively influences women’s psychological well-being in Pakistan.

H_4_: Online social capital mediates the relationship between social media use and women’s psychological well-being in Pakistan.

**Fig. 1:**
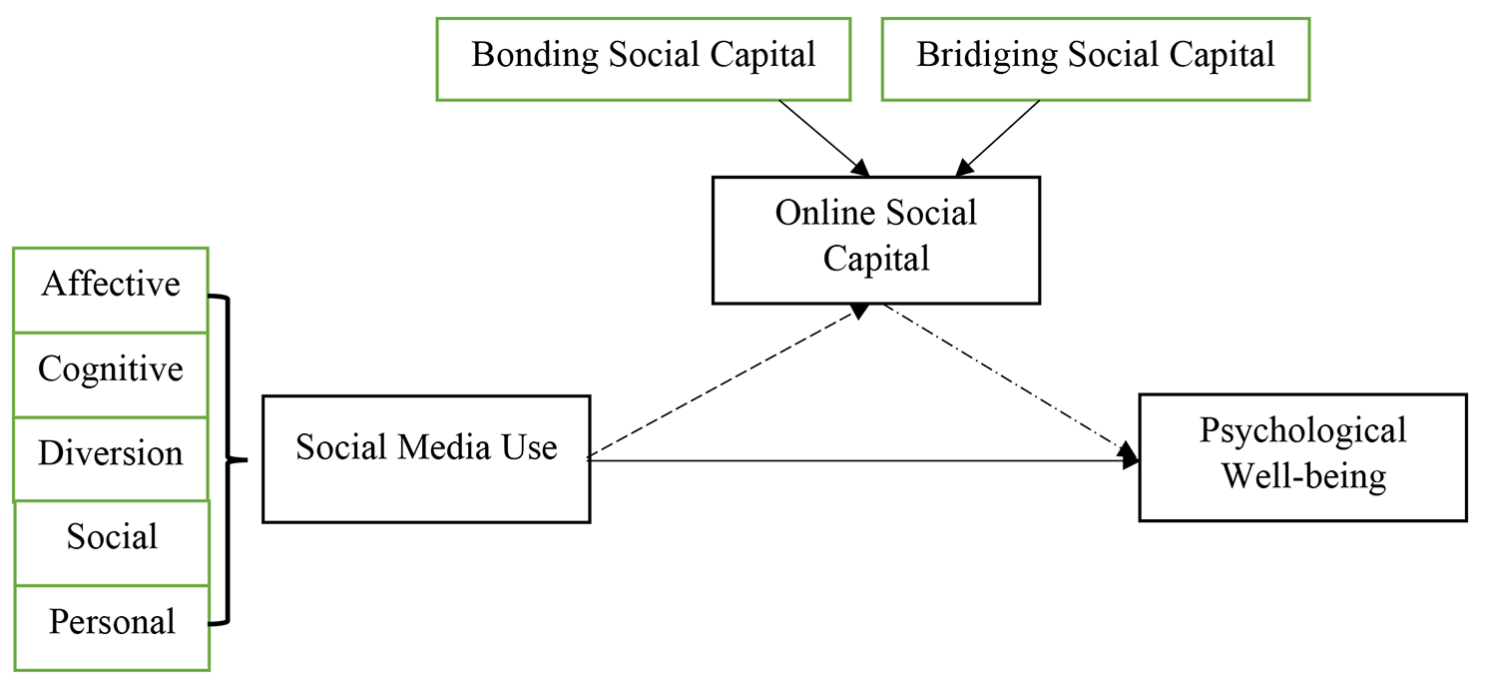
Conceptual Framewok

In line with the literature review and proposed hypotheses, we come up with our conceptual framework as shown in Fig. 1.

## Methodology

This study adopted a quantitative research design and collected data through an online survey. The questionnaire has five sections: demographics, social media use in terms of time, access and frequency of use, social media needs, social capital, and psychological well-being. All construct items were adopted from validated literature scales and rated on a five-point Likert scale. The population of the study is “women” in Pakistan who use social media. According to Pakistan Statistics Bureau [51] total, woman population of Pakistan is 101,314,780 (almost a hundred million), which is 49% of the overall population. The snowball technique [52], where the research started with a small number of participants, and requested these participants to recommend participants, who fit the research criteria. Snowball sampling is widely used in social sciences to gain a maximum response rate. The study protocol was approved by the Universiti Malay Ethics Review Committee. Respondents’ informed consent was also obtained.

### Sample size

The study was conducted in “Islamabad,” which is the capital of Pakistan. Since the city is the capital, therefore, the population is made of diverse backgrounds, and the total population of the Federal Capital is 4.5 million (male: 53% and woman: 47%). According to the Higher Education Commission (HEC), 21 universities are situated in Federal Capital (fifteen public and six private). Therefore, all the universities, located in Islamabad, were selected for the present research. Moreover, the sample size was also cross-calculated considering the guidelines for partial least squares structural equation modelling (PLS-SEM) multivariate analysis. The sample size in this model consisted of paths (independent variables); thus, with parameters set at an α of 5%, R^2^ of 0.10, and power of 80%. However, accounting for a 20% dropout rate, missing values, and outliers, the final minimum sample size was 200. Ultimately, 240 responses were obtained, strengthening the results’ sample and statistical generalizability.

#### Measurement scales

##### Social media use

29 items related to social media use were adapted from various well-validated scales, including.

Sheldon’s [53] Motives for Facebook Use scale, Khan’s [54] Social Information Seeking scale, Asghar’s [55] Information Seeking in Facebook scale, Papacharissi and Rubin’s [56] Predictors of the Internet scale, and Yang and Brown’s [57] Motives for Using Facebook and Patterns of Facebook Activities scales. Each item was measured by using the Five-Likert scale Strong agree to disagree strongly.

##### Social capital

The current study explored the relational dimension of social capital; therefore, the well-validated Internet Social Capital scale by William [25] was adopted to measure online social capital, which had 20 items (10 for bonding capital and 10 for bridging capital).

### Psychological wellbeing

The Psychological Well-being scale by Ryff [19] was adopted with six dimensions (autonomy, environmental mastery, personal growth, positive relations, purpose in life, and self-acceptance). This 42-item scale is validated in numerous studies in various languages and cross-cultural settings.

### Data collection and analysis

Participants who gave consent to participate in the online survey were asked to click the ‘Continue’ button at the link. Afterwards, they can complete the self-administered questionnaire. The data collection took almost two months, and finally, obtained 240 responses. The researchers used SPSS statistical software (version 24) and Smart-PLS version 3.0 [58] for statistical analysis.

### Preliminary data analysis

The researchers first cleaned and screened the raw data to increase accuracy and precision [59]. Then, the performed normality test showed that all items’ skewness and kurtosis values lay between ± 2 and ± 7, respectively and were distributed normally. Additionally, the variables’ variance inflation factor (VIF) values were computed. The highest measured VIF was 1.739 for social capital and 1.786 for psychological well-being. These values are below the general cut-off of 10 and the conservative cut-off of 2.5, indicating no collinearity issues among the variables. Furthermore, the correlation coefficients of all exogenous constructs were also less than 0.8, confirming no multicollinearity.

### Demographic profile

All participants (*N* = 240) were females working, and most participants were aged between 30 and 39 (52.9%), married (45%), and had completed their Master’s degrees (56.3%). In addition, all the women participants use social media, mainly accessing social media through mobile phones. 43% of participants actively use two social networking sites, Facebook and Instagram. They check social networking sites on every notification, and 42% of participants revealed that they spent 3 to 4 h online on average.

### Structural equation modeling (SEM) results

PLS-SEM was performed to observe the indirect and direct effects of the independent variables (social media needs and social capital) or the dependent variable (psychological well-being).

### Measurement model

The measurement model assesses the relationships between the study’s latent variables and the observed variables’ reliability. The minimum suggested level of reliability is 0.7, and the minimum desirable level of average variance extracted (AVE) is 0.5 to establish convergent validity. The modified model’s items met these criteria, ensuring the reliability and convergent validity of the model. (See Table 1)

**Table 1:** Results of Reliability and Convergent Validity

Construct	Item	Initial model	Modified Model	Cronbach’s Alpha	Composite Reliability	Average Variance Extracted (AVE)
Autonomy	PWB.A1	0.739	0.743	0.843	0.895	0.682
	PWB.A2	0.87	0.875			
	PWB.A3	0.886	0.895			
	PWB.A4	-0.522	Deleted			
	PWB.A5	0.763	0.78			
Environmental Mastery	PWB.EM1	0.66	0.633	0.731	0.828	0.552
	PWB.EM2	0.834	0.816			
	PWB.EM3	0.854	0.868			
	PWB.EM4	0.597	0.623			
Personal Growth	PWB.PG1	-0.382	Deleted	0.669	0.799	0.5
	PWB.PG2	0.605	0.628			
	PWB.PG3	0.784	0.784			
	PWB.PG4	0.659	0.654			
	PWB.PG5	0.762	0.75			
Purpose in Life	PWB.PL1	0.87	0.871	0.756	0.844	0.581
	PWB.PL2	0.563	0.557			
	PWB.PL3	0.75	0.751			
	PWB.PL4	0.829	0.831			
	PWB.PL5	0.012	Deleted			
Positive Relations	PWB.PR1	0.752	0.724	0.729	0.832	0.555
	PWB.PR2	0.868	0.857			
	PWB.PR3	0.664	0.701			
	PWB.PR4	-0.428	Deleted			
	PWB.PR5	0.652	0.687			
Self-Acceptance	PWB.SA1	0.801	0.799	0.775	0.857	0.602
	PWB.SA2	0.839	0.839			
	PWB.SA3	0.799	0.798			
	PWB.SA4	0.651	0.654			
Bonding Capital	SC.Bonding1	0.843	0.852	0.932	0.946	0.746
	SC.Bonding2	0.867	0.865			
	SC.Bonding3	0.857	0.864			
	SC.Bonding4	0.823	0.828			
	SC.Bonding5	0.893	0.896			
	SC.Bonding6	0.477	Deleted			
	SC.Bonding7	0.877	0.875			
Bridging Capital	SC.Bridging1	0.766	0.768	0.881	0.908	0.584
	SC.Bridging2	0.756	0.758			
	SC.Bridging3	0.798	0.796			
	SC.Bridging4	0.72	0.717			
	SC.Bridging5	0.786	0.783			
	SC.Bridging6	0.773	0.775			
	SC.Bridging7	0.747	0.749			
Affective Use	SM.A1	0.688	0.687	0.813	0.887	0.727
	SM.A2	0.909	0.91			
	SM.A3	0.939	0.939			
Cognitive Use	SM.C1	0.781	0.779	0.795	0.866	0.618
	SM.C2	0.733	0.733			
	SM.C3	0.86	0.86			
	SM.C4	0.765	0.767			
Diversion Use	SM.D1	0.838	0.838	0.78	0.871	0.693
	SM.D2	0.832	0.831			
	SM.D3	0.826	0.828			
Personal Use	SM.P1	0.907	0.908	0.928	0.949	0.823
	SM.P2	0.925	0.925			
	SM.P3	0.916	0.917			
	SM.P4	0.88	0.879			
Social Use	SM.S1	0.756	0.759	0.832	0.88	0.596
	SM.S2	0.716	0.713			
	SM.S3	0.843	0.843			
	SM.S4	0.705	0.701			
	SM.S5	0.832	0.833			

### Discriminant validity

The discriminant validity assessment was measured using the Heterotrait-Monotrait ratio of correlations (HTMT). All the obtained values are less than 0.85 or 0.90, and all constructs met the discriminant validity requirement.

### Structural model

As per the research hypotheses, the structural model evaluated the effect of the five components of social media use (diversion, cognitive, affective, personal, and social) and the two subscales of online social capital (bonding social capital and bridging social capital) on psychological well-being using the bootstrapping approach.

First, the statistical analysis confirms that social media needs positively and significantly impacted online social capital, except for cognitive and personal needs on bridging capital and social needs on bonding capital. The direct effect of all five social media needs on psychological well-being was statistically significant. And lastly, online social capital, with both dimensions, bonding and bridging social capital, were found to have a positive and significant impact on psychological well-being. (see Table 2)

**Table 2:** Path Model Results

Path	β	SE	*t*-value	*p*-value
Path A				
DIV Use -> BO	0.201	0.078	2.58**	0.010
DIV Use -> BR	0.434	0.063	6.838**	< 0.001
COG Use -> BO	0.183	0.067	2.707**	0.007
COG Use -> BR	0.024	0.075	0.319	0.750
AFF Use -> BO	-0.384	0.071	5.414**	< 0.001
AFF Use -> BR	-0.142	0.063	2.237*	0.025
PER Use -> BO	0.266	0.09	2.964**	0.003
PER Use -> BR	0.068	0.068	0.999	0.318
SOC Use -> BO	-0.058	0.064	0.909	0.363
SOC Use -> BR	0.32	0.054	5.904**	< 0.001
Path B				
BO-> PWB	0.16	0.048	3.292**	0.001
BR-> PWB	0.275	0.066	4.134**	< 0.001
Path C				
DIV Use -> PWB	0.221	0.071	3.118**	0.002
COG Use -> PWB	0.246	0.054	4.554**	< 0.001
AFF Use -> PWB	-0.256	0.058	4.426**	< 0.001
PER Use -> PWB	-0.274	0.064	4.299**	< 0.001
SOC Use -> PWB	0.134	0.054	2.463*	0.014

### Mediation analysis

The mediating effect of social capital (Bonding and bridging) was analyzed to evaluate the direct and indirect effects of social media use on psychologicalwellbeing. According to the results in Table 3, both the direct and indirect effects of social media use on psychologicalwellbeing were found statistically significant. The results thus confirm that bonding social capital partially mediates the link between social media use (except for social needs) and psychologicalwellbeing, while bridging social capital only mediates the link between socialization and well-being.

**Table 3:** Total (Direct and Indirect) Effects

Path	Mediator	Total	Direct	Indirect	Result
**DIV◊PWB**	BO	0.373** (< 0.001)	0.221** (0.002)	0.032* (0.047)	Partial mediation
	BR			0.119 (0.00)	No mediation
**COG◊PWB**	BO	0.281** (< 0.001)	0.246** (< 0.001)	0.029* (0.045)	Partial mediation
	BR			0.007 (0.754)	No mediation
**AFF◊PWB**	BO	-0.357** (< 0.001)	-0.256** (< 0.001)	-0.061* (0.004)	Partial mediation
	BR			-0.039 (0.051)	No mediation
**PER◊PWB**	BO	-0.213** (< 0.001)	-0.274** (< 0.001)	0.042* (0.022)	Partial mediation
	BR			0.019 (0.305)	No mediation
**SOC◊PWB**	BO	0.213** (< 0.001)	0.134* (0.014)	-0.009 (0.401)	No mediation
	BR			0.088** (0.001)	Partial Mediation

* *Significant at 0.05 level*

** *significant at 0.01 level*

### Analysis

This study studied social media’s impact on Pakistani women’s psychological well-being through social capital. The data showed that most Pakistani women use social media, demonstrating the spread of new media in emerging nations and among women. Furthermore, despite Pakistan’s communal and patriarchal society, where women’s status is ignored, the current study shows the exponential rise of social media among women, indicating a technical change among low-income women [60].

Women’s social media usage is comparable to developed nations, suggesting that women’s online activity is rising. Statistics show that women use social media for entertainment and socializing. These data also show Pakistani women’s needs and place in a culture with few public interaction choices and social media use. These results affirm participants’ societal and gender status and support that gender dominates social media use. The marginalization of women limits their freedom, and social media helps them escape their hard daily lives. Our findings support recent research that cellular-based social media apps, high-speed Internet, and low-cost mobile phones in Pakistan have increased social media use [39]. However, gender and culture influence their social media use.

In the study of social media use and social capital, the data values of bonding and bridging social capital show that cultural forces are enforced, and women follow cultural norms online. With bridging, cognitive use is not statistically important, indicating that women can only learn from known partners. Online sources are untrustworthy. Women do not reveal their identities to strangers on social media, either. These results show the societal trap for women using digital tools and setting virtual limits. This supports societal repetition in virtual interactions. In autocratic societies, women use social media to extend their online contact (bridging social capital) for escape due to limited IRL opportunities. Socializing—especially with men—is frowned upon. Women are using social media at home to relax and release stress from their busy lives, domestic duties, and social roles as wives, daughters or mothers. Women want to defy societal norms by meeting new people and forming online relationships.

The data suggest that social media dramatically affects psychic health. However, the mediation analysis again shows the gender placement of individuals in a societal setting where women have few friends (bonding social capital) and the varying effects of bridging and bonding social capital on psychological health. Bridging capital does not impact cognitive, diverting, emotional, personal use, or psychological health. This shows that women do not use expanded online networks for information, identity, or escape. This hurts their mental health. Online socialization and social wealth affect women’s mental health.

## Discussion

Pakistani women are prone to severe violence and psychiatric illnesses because social and societal structures limit their freedom and assimilation [61]. In addition, despite societal and gender differences, social media affects women’s mental health. Considering multiple factors, including the lack of women’s inclusion in new media research in emerging economies and specifically in patriarchal societies, where women suffer more psychological disorders, the study was conducted to fill this academic gap and aimed at exploring social media effects on women’s psychological well-being and social capital.

Previous studies [62] focused on young people of both genders, for example, Ellison et al. [63] found no significant relationship between gender and Facebook use in predicting social capital, and Valenzuela et al. [64] similarly found that gender did not affect associations between Facebook use and social capital variables. Contrary to these studies, Antonio and Tuffley [65] described gender as an important factor in using social media and found more women social media users as compared to men. The research focusing on women and online social capital has revealed that women tended to experience more bonding social capital than men. Similarly, a recent study found a lower level of online bridging social capital among women due to their careful selection of online friends and not perceiving the benefits of weak ties [66]. Women spent excessive time online; however, they experience a loss of online social capital [78]. As shown in the findings, the concept of online social capital, which encompasses bonding and bridging social capital, can play a role in influencing women’s psychological well-being in Pakistan. Online platforms and social networking sites can facilitate the formation of bonding social capital by connecting women with their friends, family, and support networks. Through these connections, women can receive emotional support, share experiences, and find a sense of belonging, which can positively impact their psychological well-being. Also, the development of bridging social capital connects women from different backgrounds, regions, and cultures. This diversity of connections fosters social integration, promotes understanding, and expands social networks. By interacting with a broader range of individuals, women can gain new perspectives, challenge stereotypes, and increase their social awareness, leading to enhanced psychological well-being.

There are some theoretical implications. Due to these inconsistent findings, the current findings are hopeful for the present academic debate on social media and psychological well-being in emerging countries. The present study focused on women, who are more susceptible to mental illness and lack access to care [7]. This study supports women’s use of new media tools because low-income nations like Pakistan have more dangerous socio-economic, societal, and political structures. Also, this study finds the mediating role of online social capital, which provides emotional support, access to information, empowerment, social integration, peer support, and opportunities for advocacy. Leveraging online platforms and networks effectively can help create a supportive and empowering environment for women, ultimately improving their psychological well-being.

Managerially, women use social media to amuse themselves, gain knowledge, find mental support, express themselves, and interact because male society denies their basic needs. Our findings suggest that social media can help women in patriarchal cultures meet their basic needs and better their mental health by addressing the denial of their needs in Pakistani society.

The findings confirm earlier research that escapes bring delight and well-being [45]. In addition, expression and emotional openness online promote connection [67] and good interactions [63] indicators of psychological well-being. Pakistani society is communal, with tight family ties between generations [68]. Pakistanis depend on close friends and family for material, mental, and social help. Despite many women getting education and jobs in multiple sectors, honour murders often involve women blamed for having undesirable relations outside their family network.

Despite these societal hurdles, this study shows that social media has changed and increased social capital for Pakistani women. The results are essential for women and culture because they demonstrate how technologies can change communication and engagement patterns in a strict and typically communal society like Pakistan in the digital age of virtual communities.

Gender has been vital in media choice and online social capital studies. The current study suggests that Pakistani women, who are culturally excluded, may profit from new media tools. Previous studies [69] found that women enjoy involvement and interest both within and outside the family. In a male-dominated culture, social media can help women build social capital. Indeed, despite Pakistani women’s limited sociability, they use technology to interact across regional and cultural borders and bridge their social capital to gain knowledge, build relationships, and expand their vision. Despite an adverse online climate, women use social media to build networks and connections.

Actively using social media grows women’s online social capital, which extends their networks, trust, and support and contributes to their psychological well-being. The findings support the idea that online social capital enhances and expands relationships and improves life quality and well-being.

## Conclusion

The present study posits online social capital as an important mediating variable between Pakistani women’s social media use and psychological well-being. The results are consistent with studies conducted, highlighting the importance of further exploration of gender-based social media use in varying cultural contexts. Furthermore, considering the social marginalization of Pakistani women, the results recommend that women use social media to seek support, participate in online communities, and integrate relationships to expand social capital, which consequently contributes to their psychological well-being. This stuy also contains few limitations. Firstly, he study only focused on educated women who are working in universities and are currently living in the capital of Pakistan, Islamabad. Future stusies should recruit more diversified participants and increase the generalizability of this study. Although women are working in other fields of life as well, however, the study only focused on women working in an academic position in institutions and universities in Pakistan. Secondly, this study only focuses on social media use and online social capital in determining psychological wellbeing. There might be more determinants and future studies should explore from here.

## Data Availability

The datasets generated and/or analyzed during the current study are not publicly available but are available from the corresponding author on a reasonable request.
